# Exploring the midgut physiology of the non-haematophagous mosquito *Toxorhynchites theobaldi*

**DOI:** 10.1098/rsob.230437

**Published:** 2024-07-03

**Authors:** Renata C. Barbosa, Raquel S. M. Godoy, Priscila G. Ferreira, Tiago A. O. Mendes, Marcelo Ramalho-Ortigão, José M. C. Ribeiro, Gustavo F. Martins

**Affiliations:** ^1^ Departamento de Biologia Geral, Universidade Federal de Viçosa, Viçosa, Minas Gerais 36570-900, Brazil; ^2^ Fundação Oswaldo Cruz, Instituto René Rachou, Fiocruz, Belo Horizonte, Minas Gerais 30190-002, Brazil; ^3^ Departamento de Bioquímica e Biologia Molecular, Universidade Federal de Viçosa, Viçosa, Minas Gerais 50670-900, Brazil; ^4^ NextGen Colorado Consulting LLC, Fort Collins, CO 80525, USA; ^5^ Section of Vector Biology, Laboratory of Malaria and Vector Research, National Institute of Allergy and Infectious Diseases, Rockville, MD 20852, USA

**Keywords:** digestion, evolution, innate immune response, midgut, non-haematophagous mosquito

## Abstract

*Toxorhynchites* mosquitoes have an exclusively phytophagous feeding habit as adults, which leads to significant differences in their morphophysiology compared with haematophagous mosquitoes. However, the molecular mechanisms of digestion in this mosquito are not well understood. In this study, RNA sequencing of the posterior midgut (PMG) of the mosquito *Toxorhynchites theobaldi* was undertaken, highlighting its significance in mosquito digestion. Subsequently, a comparison was made between the differential gene expression of the PMG and that of the anterior midgut. It was found that the most abundant proteases in the PMG were trypsin and chymotrypsin, and the level of gene expression for enzymes essential for digestion (such as serine protease, α-amylase and pancreatic triacylglycerol lipase) and innate immune response (including catalase, cecropin-A2 and superoxide dismutase) was like that of haematophagous mosquitoes. Peritrophin-1 was detected in the entire midgut, with an elevated expression level in the PMG. Based on our findings, it is hypothesized that a non-haematophagic habit might have been exhibited by the ancestor of *Tx. theobaldi*, and this trait may have been retained. This study represents a pioneering investigation at the molecular level of midgut contents in a non-haematophagous mosquito. The findings offer valuable insights into the evolutionary aspects of feeding habits in culicids.

## 1. Introduction

The females of haematophagous mosquitoes (Culicidae) are known to be noteworthy vectors of disease. Blood feeding is essential for mosquito females to develop and lay eggs. However, there are three non-haematophagous genera, namely, *Malaya*, *Topomya* and *Toxorhynchites*, which do not require blood to reproduce [1]. *Toxorhynchites* is the only representative of the Toxorhynchitini tribe, and it has a wide geographic distribution, found in both tropical and temperate areas [2]. The larvae of *Toxorhynchites* are predators that primarily feed on aquatic invertebrates, including mosquitoes. Adult *Toxorhynchites* feed only on nectar and plant exudates [3], not being associated with pathogen transmission. Nevertheless, cell cultures derived from several *Toxorhynchites* species are widely used in the study of arboviruses that infect vector mosquitoes, as they facilitate virus propagation and isolation *in vitro* [4]. Moreover, the predatory behaviour of *Toxorhynchites* larvae makes them a potential biological control agent against larvae of other mosquito species [5].

The mosquito midgut is a crucial site for various functions, including nutrient digestion and absorption, protein synthesis and secretion and serving as one of the most active tissues in the insect’s innate immune response [6]. In haematophagous mosquitoes, such as *Aedes*, *Culex* and *Anopheles*, the midgut is divided into two distinct sections. The first is a thin and short segment, commonly referred to as the anterior midgut (AMG), while the second is an expandable, sac-like portion called posterior midgut (PMG) [7]. The AMG of haematophagous mosquitoes is rich in carbohydrate digestive enzymes, heme-binding proteins and antimicrobial peptides (AMPs) such as cecropin and defensin [8,9]. In contrast, PMG is predominantly abundant in peptidases, with negligible expression of AMPs [9].

**Figure 1.**
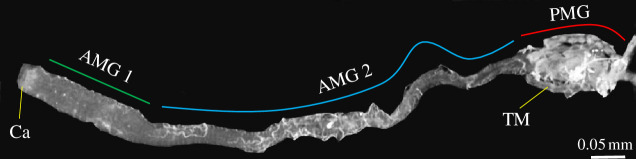
Midgut of adult female of *Tx. theobaldi* fixed with 2.5% glutaraldehyde. The anterior midgut is well developed and subdivided into anterior midgut 1 (AMG 1), located in the thorax, and anterior midgut 2 (AMG 2), located in the abdomen. The posterior midgut (PMG) is atrophied. Ca, cardia; TM, Malpighian tubule.

The midgut of adults of *Toxorhynchites theobaldi* is divided into three distinct sections: AMG1 (anterior midgut 1), located in the thorax and characterized by its short and slender shape and surface folds; AMG2, a long and slender section without folds situated in the abdominal region; and PMG, a more dilated section located in the abdomen but shorter than either AMG1 or AMG2 (figure 1). Unlike adult haematophagous culicids, which synthesize the peritrophic matrix (PM) in the PMG after a blood meal [10,11], the PM of *Tx. theobaldi* is synthesized constitutively, covering the entire midgut lumen of adults [12]. These remarkable differences between the midgut of female *Toxorhynchites* and haematophagous mosquitoes suggest the existence of a non-haematophagous condition of the ancestral into Culicidae. Currently, understanding the adaptive evolution of mosquitoes to haematophagy is challenging owing to the lack of established biological models and the limited fossil record of ancestors [13].

The feeding habits of mosquitoes have long been a topic of interest in the field of public health, particularly as it relates to disease transmission in humans and animals. This manuscript used RNA-seq to examine the global gene expression in the PMG of *T*x*. theobaldi*, the midgut region where digestion is generally more pronounced in mosquitoes. Our study also included real-time reverse transcription quantitative polymerase chain reaction (RT-qPCR) considering the three midgut sections (i.e. AMG 1, AMG 2 and PMG) separately and measurements of digestive enzyme activities to provide a more comprehensive understanding of the digestion. Further, the elucidation of the mechanisms of digestion in non-haematophagous mosquitoes expands the knowledge about the evolution of the feeding habits of mosquitoes.

## 2. Material and methods

### 2.1. Mosquitoes

The immature stages of *Tx. theobaldi*, including larvae in different instars and pupae, were collected at Mata do Paraíso in Brazil. Mata do Paraíso is a fragment of the Atlantic Forest that belongs to the Universidade Federal de Viçosa (UFV). Immature stages of this mosquito were collected year-round by placing 5 l plastic buckets filled with dechlorinated tap or rainwater on the ground near tree trunks. The insects were identified in the field based on morphological characteristics of the immature stages and then transferred to the insectarium of the Department of General Biology at the UFV without establishing a colony, with periodic collections carried out in the field. Each mosquito was placed in an individual, transparent plastic cup containing 50 ml of tap water without chlorine. The species were confirmed after adult emergence.

The larvae and pupae of *Tx. theobaldi* were identified using dichotomous identification keys [14,15] under a stereomicroscope. The larvae of *Tx. theobaldi* were daily fed with *Aedes aegypti* larvae (strain PPCampos, Campos dos Goytacazes), which are routinely maintained in our insectary, in addition to other mosquito larvae (unknown species) collected from the same plastic buckets from which *Tx. theobaldi* was collected.

The pupae of *Tx. theobaldi* were kept in plastic cups inside plastic cages (BugDorm Store, Taichung, Taiwan). After emerging, adults were provided with a 10% sucrose solution for feeding. A sugar solution was administered to the adults as their primary sustenance in their natural habitat involves nectar consumption [16]. Consequently, a diet similar to that commonly used for colonized mosquitoes was chosen for them. Both the immature stages and adults of *Tx. theobaldi* were kept at room temperature (24 ± 4°C) and under a natural light/dark cycle.

### 2.2. RNA extraction, library preparation and sequencing

The PMG (figure 1) of 8 adult female *Tx. theobaldi*, aged 7–14 d after emergence, were dissected in phosphate-buffered saline (PBS) pH 7.6 solution (0.1 M NaCl, 20 mM KH_2_PO_4_ and 20 mM Na_2_HP_4_), using scissors and pins that were decontaminated with RNAse AWAY (Sigma-Aldrich, Buchs, Switzerland) and immediately transferred to TRIzol (Invitrogen, Carlsbad, CA, USA). The PMG was then homogenized and stored at −80°C. Total RNA was subsequently isolated using phenol/chloroform from each of the two PMG pools (four samples per pool), and the RNA quality was verified on a 1% denaturing agarose gel containing 1× MOPS (Sigma M1254) (MOPS 20 mM; 5 mM sodium acetate; 1 M ethylenediaminetetraacetic acid pH 7.0 in 0.1% diethyl pyrocarbonate (DEPC)-treated water and 5% formaldehyde.

RNA integrity was assessed using the Agilent 2100 Bioanalyzer with RNA 6000 Nano chips (Agilent, Waldbronn, Baden-Württemberg, Germany) at Integrated Genomics Facility Department of Plant Pathology, Kansas State University (Manhattan, KS, USA). The quantification of the samples was performed using the Qubit 2.0 Fluorometer (Thermo Fisher Scientific), and their purity (measured at 260/230/280 nm) was evaluated using the NanoDrop ND-1000 spectrophotometer (Thermo Fisher Scientific).

High-quality total RNA (400 ng) was used for the preparation of RNA-seq libraries, following the manufacturer’s recommendations for the TruSeq RNA Sample Preparation v.2 kit (Illumina). To ensure the absence of deoxyribonucleic acid (DNA) contamination, the libraries were evaluated using the Agilent 2100 Bioanalyser with the Qubit dsDNA BR Assay kit (Thermo Fisher Scientific). Subsequently, the libraries were normalized to 10 nM in equal volumes and divided into 2 libraries as technical replicates per pool. The Illumina MiSeq platform with a MiSeq reagent v3 kit (Illumina) was used for sequencing, with a total of 600 cycles, and paired-end sequencing was performed at KSU’s Integrated Genomic Facility. The average read length was 300 bp.

### 2.3. RNA-seq analysis

For transcriptome analysis of PMG, reads with low quality (<20 bp) were trimmed, and adapter primer sequences were removed from Fastq files. The trimmed reads were then concatenated and assembled in a single-ended mode using Abyss [17] and Soap *de novo*-Trans [18] software, with a k-parameter set between 21 and 91 in increments of 5 [19]. A 3' prime poly-A enrichment was performed during the assembly process. The resulting fasta-generated files were further assembled using a BLAST and CAP3 pipeline [20]. Coding sequences (CDS) were extracted based on the presence or absence of various signatures, including signal peptides, furin cleavage sites, mucin-type O-glycosylations and transmembrane domains. Similarities with related proteins from Diptera deposited in the National Center for Biotechnology Information (NCBI) database, as well as information from Gene Ontology, KOG, Pfam, SMART, SwissProt and RefSeq (invertebrate) were used for automated annotation of the proteins. Reads were mapped to the contigs using BLASTN with a word size of 25, allowing for one gap, and a minimum identity threshold of 95%. Three different CDS extensions (.pep, .cds and .gff3) were used for mapping, with preference given to the extension with the highest score value. The expression levels of CDS were quantified using the RPKM (reads per kilobase per million reads mapped) metric. Additionally, a comparison of transcript expression was made using the ‘expression index’ which was defined as the number of reads mapped to a specific CDS multiplied by 100 and divided by the highest number of reads mapped to any CDS [21]. The ‘relative maximum RPKM’ (rmRPKM) index was also used as an expression comparison metric. Raw reads have been deposited in the NCBI Sequence Read Archive under BioProject ID: PRJNA983392, BioSample ID: SAMN35732976 and SRA ID: SRR25005524. Accession number GKNE01000001. The reads can be downloaded from https://www.ncbi.nlm.nih.gov/sra/PRJNA983392.

### 2.4. RNA extraction and RT-qPCR analysis

Total RNA was extracted from three different regions of the midgut (AMG1, AMG2 and PMG), separately. The extraction was performed on females aged 7–14 d after emergence, using TRIzol following the manufacturer’s guidelines. The concentration of total RNA was determined using a NanoDrop Lite Spectrophotometer (Thermo Scientific). For reverse transcription, 500 ng of RNA was treated with DNAse I Amplification Grade (Invitrogen) and then used in a reverse transcription reaction with the SuperScript III Reverse Transcriptase (Invitrogen). The RT-qPCR amplification was carried out using the Eco Real Time PCR System (Illumina) and the GoTaq qPCR Master Mix (Promega). The RT-qPCR reaction involved activating the enzyme at 95°C for 2 min, followed by 40 cycles of denaturation at 95°C for 5 s, and annealing/extension at 60°C for 30 s.

The relative expression levels of the target genes (table 1) were calculated using the 2^−ΔΔCT^ method [22] and analysed using the one-way ANOVA test followed by the Tukey test for multiple comparisons of means. GraphPad Prism 7.00 software for Windows (La Jolla, CA, USA, www.graphpad.com) was used for statistical analysis. All tests were considered significant at *p* ≤ 0.05. Three independent biological samples were used for each region of the midgut (AMG1, AMG2 and PMG), and the housekeeping gene (ribosomal protein S17, RPS17) was used as a reference gene. The genomic DNA of the PMG was used as a calibration sample to determine the ΔCt (cycle threshold).

**Table 1.** List of genes and primers used for RT-qPCR analysis.

gene	acession N°	primers 5′−3′		product size (pb)
		forward	reverse	
reference gene				
40S ribosomal protein S17 (RPS17)	Tt-39953_FR5_13-155	CAGCTCATTTGCTGGTTTCA	CTTGCTGGCTGTTTGTTCAG	99
target genes				
*α*-amylase	Tt-28334_FR3_60-659	CAGCTCATTTGCTGGTTTCA	CTTGCTGGCTGTTTGTTCAG	99
second ferritin light chain subunit (LCH2)	TT-28334_FR3_60-659	AATCTCCGACAAAGCCTGGG	GGCACCACGTTTGCTTTGAT	98
peritrophin-1	TxSigpSigP-30727_FR4_217-393	CGATCCGTTGATTGCCGTTC	CCACTAGCTGACCCCAACTG	98
serine protease	Tt-62962_FR4_136-382	CACTGGTTTGCCCGATACCT	ATACCTTCTTGTCCGCAGCC	95
catalase	Tt-25992_FR1_55-581	TCGCCAACCATCTGAGCAAT	CTTCGGACAGTTGACGTCCA	100
cecropin-A2	TxSigpSigP-65655_FR5_25-87	GCTGAAGAAACTCGGCAAGA	CCGATCGCTTTATAACCAGC	92
Cu2+/Zn2+superoxide dismutase	Tt−62593_FR5_49-217	CTGAAAGCAGGCAACCATGG	TTGCCGTGTGGGTTGAAATG	97
pancreatic triacylglycerol lipase (PTL)	TxSigpSigP-23674_FR4_7-348	ATTGGTCATAGCCTGGGTGC	CCCACTGAGAAGAACGGACC	99

### 2.5. Assay for total protein content and assay for trypsin activity

Ten adult females, aged 7–14 d, were reared as previously described. The whole midguts (figure 1) were collected and homogenized in 100 µl of PBS on ice, using Pellet tissue pestles (Sigma, cat. num. Z359971). The homogenate was then centrifuged at 10 000 rpm for 5 min at 4°C using an Eppendorf centrifuge (model: 5418R). The resulting supernatant was used for testing trypsin activity, determining nitric oxide (NO) levels and measuring total protein concentration. Trypsin activity was measured using N-alpha-benzoyl-DL-arginine-to-nitroanilide (BApNA) (Sigma, cat. num. B4875; St. Louis, MO, USA) as a substrate, at a final concentration of 0.36 mM in a buffer solution consisting of 0.1 M Tris-HCl and 20 mM CaCl_2_, pH 8.0.

The trypsin activity assay was performed by incubating 20 µl of the supernatant (midgut extract) and 30 µl of 1.2 mM BApNA. The absorbance (405 nm) was measured after 10 min at 25°C using a plate reader (Mulitskan GO, Thermo Fisher Scientific). Two controls were prepared under the same conditions, in the absence of the supernatant or the enzyme. Enzymatic activity was expressed in units (U), defined as the amount of enzyme capable of hydrolysing 1 µmol of BApNA per minute under the specified conditions, using a molar coefficient of 8800 M^−1^ cm^−1^. Assays were performed in triplicate and repeated three times independently. The protein content of the midgut was determined using 3 µl of supernatant with the Bradford method [23], utilizing 140 µl of Bradford reagent (BioRad). Standard curves for determining albumin concentration were constructed using various concentrations of monoreagent albumin (Bioclin, K040) in triplicate assays performed in three independent experiments. Absorbance readings were measured at 595 nm using a plate reader (Mulitskan GO). Mean values ± standard deviation (s.d.) were calculated using GraphPad Prism 7.00 software for Windows.

### 2.6. Quantification of reactive oxygen species

#### 2.6.1. Nitric oxide

The NO production was analysed by incubating 10 µl of homogenate supernatant (diluted in 40 µl of buffer solution) with 100 µl of Griess reagent (0.5% sulfanilamide and 0.05% N-(1-naphthyl)-ethylenediamine in 2.5% phosphoric acid) for 10 min in the dark. Absorbance was measured at 560 nm using a plate reader [24]. NO levels were quantified using a calibration curve of NaNO_2_ (0−125 µM) and calculated as mean ± s.d. using the GraphPad Prism 7.00 software.

#### 2.6.2. Soluble peroxides

Three midguts were dissected and homogenized in 35 µl of PBS containing the catalase inhibitor (3-amino−1,2,4-triazol; Sigma-Aldrich; 2 mg ml^−1^) for each sample. The samples were then centrifuged at 15 000 g for 5 min at 4°C (Eppendorf centrifuge 5418R). Next, 10 µl of each sample were diluted 1:5 and added to 950 µl of ferrous oxidation-xylenol orange (FOX) reagent (100 mM sorbitol, 100 µM xylenol orange; 250 µM ferrous ammonium sulfate; 25 mM H_2_SO_4_) and incubated at room temperature for 30 min. The absorbance was measured at 596 nm using a plate reader (Mulitskan GO) [25]. The concentration of H_2_O_2_ was quantified using a calibration curve of pure H_2_O_2_ (0–1000 µM). The mean ± s.d. of the fluorescence intensity values from three independent experiments were calculated using the GraphPad Prism 7.00 software.

### 2.7. Phylogenetic analysis

To study molecular evolution of peritrophins, eight representative species of the order Diptera (both adult and larvae) including *Tx. theobaldi* were selected. The sequences were aligned using MAFFT (version 7) [26] with the iterative refinement method parameter E-INS-i recommended for less than 200 sequences. Consensus domains of chitin-binding proteins (ChtBD2, pfam01607) were confirmed using the NCBI CDD conserved domain database scanning program (https://www.ncbi.nlm.nih.gov/Structure/cdd/wrpsb.cgi) via BLAST search. Subsequently, the sequences were submitted to the MrBayes program (version 3.2.2) [27] for Bayesian inference through the virtual platform CIPRES (https://www.phylo.org/) [28], with the option to test the best model of data evolution during the analysis. The result showed that Whelan and Goldman (WAG) was the best model for the evolution of proteins in this study. Subsequently, the consensus tree was obtained by running a Markov chain in two series of four chains each (with 25% burning) for one million generations. The consensus tree was then visualized using the program FigTree (http://tree.bio.ed.ac.uk/software/figtree/). The files containing the alignment and script used in this study are included as supplementary data files (electronic supplementary material, file 1).

### 2.8. Analysis of orthologous

The protein sequence predicted for *Tx. theobaldi* was compared with the protein sequences of four other closely related culicid species, namely *Ae. aegypti*, *Anopheles gambiae*, *Anopheles darlingi* and *Culex quinquefasciatus*, and clustered into groups based on sequence similarity using the OrthoVenn 2.0 web server [29] (https://orthovenn2.bioinfotoolkits.net/home). The analysis involved all-to-all protein similarity comparisons with an *e*-value cutoff of 1*e* ^−5^ and an inflation value of 2.0.

### 2.9. The analysis of early trypsins

The multisequence alignment of early trypsin of the mosquistoes *Tx. theobaldi* (accession number: TxSigpSigP-22990_FR2_1-147), *Ae. aegypti* (accession number: AAEL007818), *An. gambiae* (accession number: CAA80515.1) and *Cx. quinquefasciatus* (accession number: CAA80515-1) were performed using the Clustal Omega software (https://www.ebi.ac.uk/jdispatcher/msa/clustalo) [30] and PROSCAN function of the PROSITE suite (http:// prosite.expasy.org) [31] for identification domain, active site, the signal peptide and the conserved cysteine amino acids of disulfide bonds.

## 3. Results

### 3.1. *De novo* transcriptome assembly and annotation

**Figure 2.**
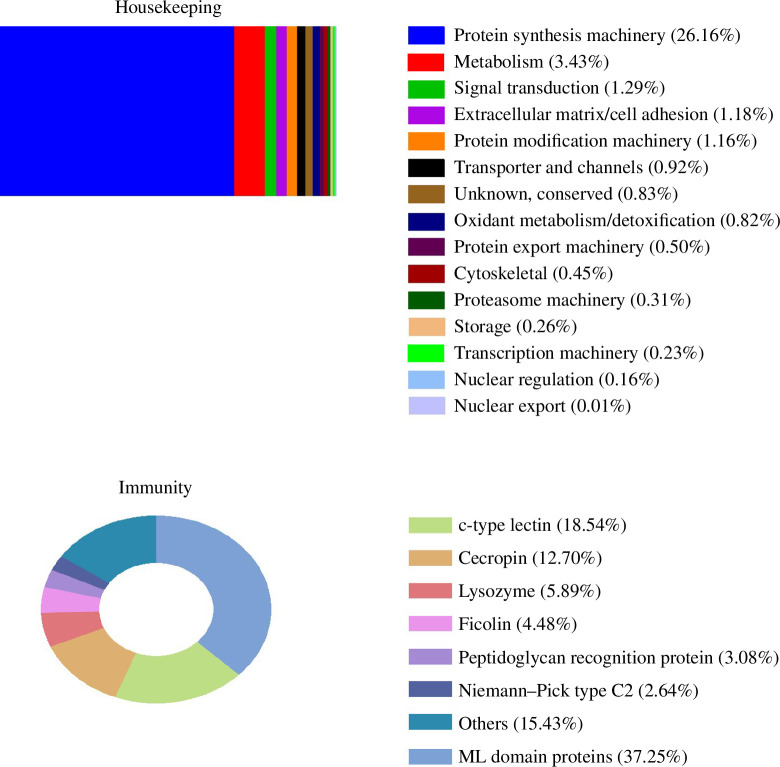
Functional classification. Abundance relative of housekeeping classes and immunity-related transcript in the PMG of adult females of *Tx. theobaldi*.

Sequencing of PMG libraries and *de novo* assembly produced 25 514 372 reads generated 17 359 contigs with an average length of 722 bp (largest contig is 7740 bp and the smallest is 150 bp) (electronic supplementary material, data sheet 2, file 2). Among these contigs, 14 060 (81%) CDS are complete. The contigs were classified in 6 classes, major categories namely secreted (4643 contigs or 35.65%), unknown (5517 contigs or 25.15%), transposable elements (TE; 195 contigs or 0.11%), viral (33 contigs or 0.03%), immunity (258 contigs or 1,28%) and housekeeping (6971 contigs or 38.37%) (figure 2; electronic supplementary material, data sheet 3, file 2). The abundance of contigs was analysed using the RPKM normalization method for each mapped CDS.

The indicator ‘relative maximum RPKM (rmRPKM)’ was established to serve as an expression index, as described in the Material and methods section. The maximum value of RPKM found was set as rmRPKM = 100, and the rmRPKM values for the remaining *n* transcripts were determined as rmRPKM(*n*) = RPKM(*n*) × 100/rmRPKM(100). We arbitrarily selected values of rmRPKM < 1 to correspond to constitutive expression, values of 1 < rmRPKM < 10 to indicate low expression levels and values >10 to indicate high expression levels (electronic supplementary material, data sheet 1, file 2). In general, all functional classes present in the PMG of *Tx. theobaldi* showed rmRPKM < 1, except for members of the protein synthesis machinery and protein-secreted classes.

The housekeeping category was identified, annotated and divided into 21 functional classes (figure 2; electronic supplementary material, data sheet 4, file 2). Notably, the protein synthesis machinery class constituted the largest group, accounting for 26% of all transcripts. In addition, several housekeeping classes, such as those involved in digestion, were thoroughly investigated owing to their critical role in biological processes (figure 2).

The class of hypothetical conserved secreted protein precursors comprised a significant portion, accounting for 71.20% of all secreted RPKM (table 2; electronic supplementary material, data sheet 1, file 2). The other two classes, mucin and protease, followed with 11.32 and 10.08%, respectively. Further analysis of the putatively identified transcripts revealed 257 mucins (table 2; electronic supplementary material, data sheet 1, file 4) that contained Pro/Ser/Thr-rich spacers and furin cleavage sites (electronic supplementary material, data sheet 1, file 2). Overall, the mucin transcripts exhibited a low rmRPKM, except for contig TxSigpSigP-22869_FR6_18-113, which corresponds to a hypothetical secreted protein precursor with an rmRPKM of 100. The most abundant transcripts in the PMG were those associated with various proteases, such as trypsin, chymotrypsin, serine protease, cathepsin, cysteine and metalloproteinase (table 2 and electronic supplementary material, data sheet 1/data sheet 4, file 2). Additionally, transcripts encoding protease inhibitors (1.83%) were identified, including serine protease inhibitors (serpins), cystatin-like proteins and proteins with Kunitz and Kazal domains. Several transcripts were also identified with putative roles in innate immunity, transcripts of antigen 5 family, odorant-binding proteins (OBPs), lectins and leucine-rich repeats (table 2; electronic supplementary material, data sheet 1/data sheet 4, file 2).

**Table 2.** Functional classification of contigs for putative secreted proteins found in the PMG of adult females of *Tx. theobaldi*.

class	contigs	reads	reads/contigs	%reads
**antigen 5**	7	3512	501.71	0.04
**hormones**	10	3821	382.1	0.04
**immune-related products**				
C-type lectin	2	5271	2635.5	0.06
galactose-specific C-type lectin	1	5599	5599	0.07
galectin	5	36 236	7247.2	0.42
hypothetical secreted protein precursor	2	3038	1519	0.04
leucine-rich repeat	5	2115	423	0.02
techylectin-5B	3	700	233.33	0.01
**lipases**	27	23 539	871.81	0.27
**lipocalin**	4	949	237.25	0.01
**mucins**	257	9 97 957	3883.1	11.62
**nucleases**	21	20 935	996.9	0.24
**OBP**	7	14 906	2129.43	0.17
**proteases**				
trypsin	25	5 36 933	21 477.32	6.25
chymotrypsin	11	1 59 620	14 510.91	1.86
M1 aminopeptidase	6	12 385	2064.17	0.14
serine protease	31	20 390	657.74	0.24
anionic trypsin-2	13	8049	619.15	0.09
trypsin-like serine protease	9	3691	410.11	0.04
cysteine proteinase	3	3261	1087	0.04
cathepsin	7	26 073	3724.71	0.3
metalloprotease	7	2646	378	0.03
others	26	93 302	3588.54	1.09
**proteases inhibitors**				
metalloproteinase inhibitor 3	2	840	420	0.01
serpin	27	17 783	658.63	0.21
trypsin inhibitor like cysteine-rich domain	5	410	82	0
cystatin-like protein	1	379	379	0
thyroglobulin typeIrepeats	6	13 436	2239.33	0.16
kazal	8	1 23 954	15 494.25	1.44
**reprolysin**	4	236	59	0
**toxin**	1	95	95	0
**conserved**				
hypothetical conserved secreted protein precursor	9	1550	172.22	0.02
**unknown**				
hypothetical secreted protein precursor	3952	6 116 648	1547.73	71.2
unknown conserved	69	55 438	803.45	0.65
**others secreted proteins**	70	2 74 918	3927.4	3.2
**total**	4643	8 590 615	1850.23	100

### 3.2. Orthology analysis

**Figure 3.**
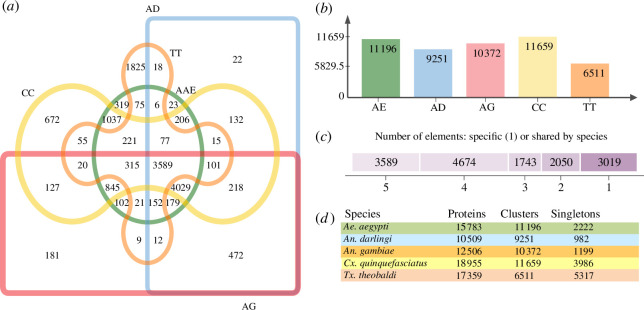
Distribution of the orthologous clusters across five species of mosquitoes. (*a*) Venn diagram showing the distribution of the orthologous clusters across five species of mosquitoes (AA = *Aedes aegypti*, AD = *Anopheles darlingi* AG = *Anopheles gambiae,* CC = *Culex quinquefasciatus* and TT = *Toxorhynchites theobaldi*). (*b*) The numbers refer to the distribution of all the orthologous clusters in mosquitoes. (*c*) Distribution of the number of elements present in shared orthologous clusters among different species. (*d*) Summary of the number of proteins, clusters and singletons deduced from *Tx. theobaldi* PMG compared with the proteome of other species.

Orthologous gene clusters were constructed between *Tx. theobaldi* and several haematophagous culicid species, including *Ae. aegypti*, *An. darlingi*, *An. gambiae* and *Cx. quinquefasciatus*, using OrthoVenn. A total of 13 348 orthologous clusters, consisting of 3589 genes, were found to be shared among these species (figure 3), with 1637 of these identified as single-copy gene clusters. Furthermore, 1825 unique clusters were encoded by *Tx. theobaldi*, with functions associated with oxidoreductase activity, an integral component of membrane, innate immune response, trehalose transport and DNA-mediated transposition. Additionally, 5317 singleton proteins were identified. Based on the high number of unique clusters and singleton expansions, it can be hypothesised that *Tx. theobaldi* is more distantly related to other mosquito species.

### 3.3. Identification, phylogenetic analysis, and expression analysis of peritrophins

The transcriptome of PMG yielded a total of six peritrophins, all of which contained the peritrophin-A domain. The classification of peritrophins is based on the composition of their chitin-binding domain (CBD) and mucin domain (MD) into four types, which can influence their function in the PM [32]. In *Tx. theobaldi*, peritrophins were found to be simple, containing only 1–2 CDBs, and at least one MD may be present.

**Figure 4.**
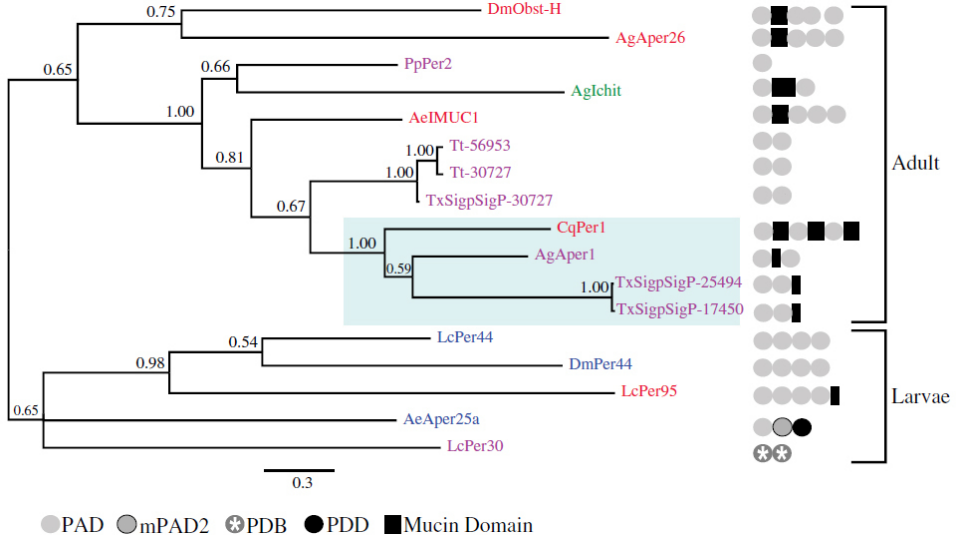
Phylogenetic tree of peritrophins. The tree was constructed using Bayesian inference of peritrophins in eight species of dipterans: *Ae. aegypti* (Ae), *An. gambiae* (Ag), *Cx. quinquefasciatus* (Cq), *Drosophila melanogaster* (Dm), *Lucilia cuprina* (Lc)*, Phlebotomus papatasi* (Pp) and *Tx. theobaldi* (Tx/Tt) with MrBayes 3.2.6 software implemented in CIPRES [28] based on the sequences aligned with MAFFT version 7. The values next to each node correspond to the subsequent probability of each one using the WAG (Whelan and Goldman) amino acid substitution model. The name of all proteins was obtained elsewhere [32], and the sequences were obtained from the NCBI GenBank. The colours of each protein refer to the classification in simple peritrophin (purple), binary peritrophin (green), complex peritrophin (red) and repetitive peritrophin (blue), specifying the structural organization of them to the CBDs (chitin binding peritrophin A domain, PAD; modified PAD, mPAD2; tachycitin, PDB; peritrophin-D domain, PDD; and mucin domain). The scale bar represents the number of nucleotide substitutions per site for the set of sequences analysed. The blue-coloured area highlights the peritrophin-1 cluster, which plays an important role in the immune response and maintenance of the integrity of the PM.

Phylogenetic analysis, which included eight dipteran species, revealed that duplication events occurred after speciation processes in *Tx. theobaldi* (figure 4). The peritrophins identified (five in total) were found to be similar (53−65%) to peritrophin-1 (PER1) observed in several culicids, such as *Ae. aegypti*, *An. gambiae* and *Cx. quinquefasciatus*. In particular, the contigs TxSigpSigP-25494 and TxSigpSigP-17450 of *Tx. theobaldi* (which were identified as in-paralogous) formed a sister clade with the AgAper1 peritrophin-1 of *An. gambiae* (figure 4).

The peritrophin sequences analysed from haematophagous mosquito larvae and adults exhibit multiple domains (1–4 CBDs + <3 MDs) (figure 4). However, adult females of *Tx. theobaldi* possess only a few peritrophins with limited domains (figure 4). Phylogenetic analysis and examination of peritrophin structure demonstrate that multiple duplication events occurred during the evolution of dipterans. Therefore, the notable clustering of certain sequence pairs (e.g. Tt-56953 and Tt-30727, or TxSigpSigP-25494 and TxSigpSigP-17450) suggests that these sequences could be the result of relatively recent gene duplication events.

**Figure 5.**
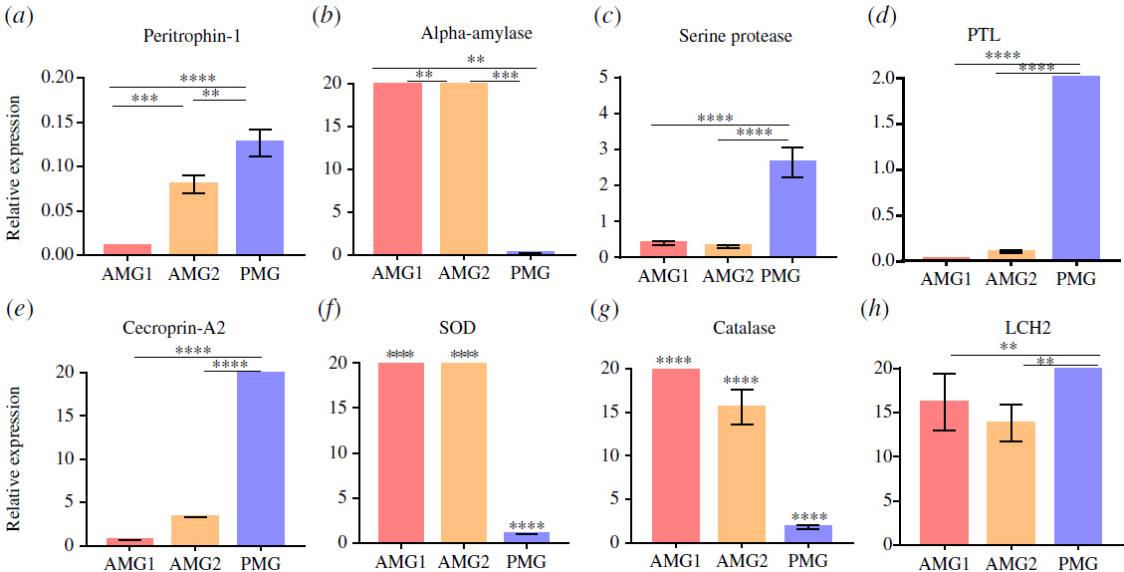
Gene expression levels of housekeeping genes secreted in the midgut of adult females of *Tx. theobaldi*. Relative expression levels of genes associated with metabolism, detoxification, immunity and PM including (*a*) peritrophin-1 (a PM protein), (*b*) *α*-amylase, (*c*) serine protease, (*d*) pancreatic triacylglycerol lipase (PTL), (*e*) cecropin-A2 (AMP), (*f*–*g*) *ROS-*detoxifying enzyme (superoxide dismutase and catalase) and (*h*) ferritin light-chain homologue (LCH2) in the three regions of midgut (AMG1, AMG2 and PMG). The experiments were carried out in triplicate, with three biological replicates. The transcriptional levels were obtained by the one-way ANOVA test followed by Tukey tests for multiple comparisons, the level of significance adopted was *p* < 0.05. Asterisks represent significant differences, and the bars indicate the mean ± s.d. The analysis was performed using the GraphPad Prism 7.00 software for Windows. Where indicated: **p < 0.05, ***p < 0.001, ****p < 0.0001. The absence of an asterisk indicates a non-significant difference.

The expression level of peritrophin-1 in the midgut of *Tx. theobaldi* was higher in the PMG when compared with AMG 1 and 2 (figure *5a*). This shows a specialization among the three midgut regions.

### 3.4. Digestive enzymes

The digestion process of *Tx. theobaldi* seems to initiate with α-amylase, which breaks down α-1,4-glycoside bonds found in complex polysaccharides such as starch derived from plant cells. Other carbohydrates are then broken down by enzymes like glucosidases, glycosyl hydrolase family 38, trehalase and β-mannosidase, all of which were identified in the mosquito’s transcriptome (electronic supplementary material, file 2). Carbohydrate digestion occurs within the mosquito’s PMG (table 2; electronic supplementary material, data sheet 1, file 1). Within the class of carbohydrases, α-amylase accounts for the largest representation in the PMG (approx. 21% for the carbohydrate metabolism class). Considering the five different species of mosquitoes, three orthologous and four co-orthologous genes for α-amylase were identified. It also identified six in-paralogous putative genes for α-amylase in *Tx. theobaldi*. α-amylase expression is higher in the AMG and significantly lower in the PMG (figure *5b*).

**Figure 6.**
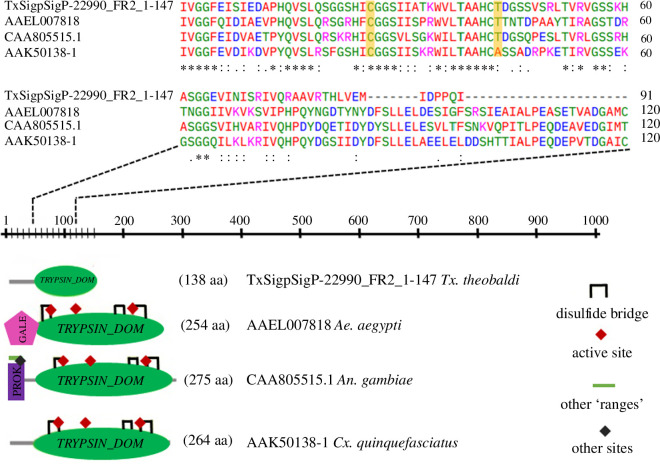
Architecture and alignment of the mosquito’s early trypsin domains. The trypsin main architecture (green) is highly conserved among the mosquitoes. Clustal and Prosite were employed for domain analysis. Conserved amino acids are marked with asterisks, and conserved cysteine residues involved in disulfide bonds are highlighted with yellow boxes. GALE, galectin; PROK, prokar lipoprotein.

The PMG synthetizes 124 transcripts encoding various types of digestive proteinases, such as cathepsins (B and l), chymotrypsin (1, 2 and BI), metalloproteinase, serine protease, trypsin-like serine protease and trypsin (1, 4, delta/gamma, eta and epsilon). The majority of these transcripts were found to be present at basal levels (rmRPKM < 1). Serine proteases, which are crucial digestive enzymes, constitute 75% of the enzymes secreted into the lumen and are present throughout the midgut with higher concentrations in PMG (figure *5c*). Putative trypsin proteins were identified as in-paralogs, as the trypsin-1 and trypsin-4. The analysed trypsin-4 sequence was consistent with conserved trypsin domains to Antryp4 of *An. gambiae* and early trypsins of *Ae. aegypti* and *Cx. quinquefasciatus*, which are constitutively expressed in the midgut of adult female mosquitoes (figure 6). Additionally, we assessed the total protein content using Bradford reagent and detected trypsin activity through the hydrolysis of BapNa in the entire midgut of adult female *Tx. theobaldi*. The protein levels were 1.548 ± 0.03606 µg per midgut, and trypsin activity was 0.6031 ± 0.333 mU per midgut.

Pancreatic triacylglycerol lipase (PTL) plays a crucial role in lipid catabolism in the PMG of *Tx. theobaldi* and accounts for approximately 22% of the key enzymes involved in this process. PTL is highly expressed in the PMG region (figure *5d*), which also exhibits a significant expression of transcripts that encode other digestive lipases, including esterases/lipases, acid sphingomyelinase/PHM5 phosphate metabolism protein and triglyceride lipase-cholesterol esterase (electronic supplementary material, data sheet 4, file 2). The PMG region also contains intracellular proteins that exhibit an affinity for fatty acids, such as fatty acid-binding protein and very-long-chain acyl-CoA synthetase/fatty acid transporter. This portion of the organ also expresses proteins involved in sterol and cholesterol absorption, including sterol carrier protein 2, cholesterol transport protein (Niemann-Pick C disease protein) and the cholesterol-binding START domain of mammalian START1. Moreover, transcripts involved in β-oxidation, such as 3-hydroxyacyl-CoA dehydrogenase (rmRPKM approx. 0.9), enoyl-CoA hydratases and carnitine O-acyltransferase, suggest that *Tx. theobaldi* utilizes fatty acid oxidation as an energy source.

### 3.5. Protease inhibitors

Out of the 49 protease inhibitors identified in PMG of *Tx. theobaldi*, a majority (53%) belong to the serpin class (table 2). Additionally, we found eight serine protease inhibitors with the Kazal domain, which is the second most abundant class of transcripts in this mosquito (table 2). It was also identified eight thyroglobulin and six cystatin belonging to two other classes of protease inhibitors (table 2). Interestingly, protease inhibitors were not detected in the sialome of *Toxorhynchites amboinensis* [33].

### 3.6. ROS production and antioxidant enzymes

The humoral immune response in mosquitoes primarily involves cells found in the haemolymph [34] and also includes cells in the midgut epithelia and the salivary glands [9,35]. In *Tx. theobaldi*, it was identified immune-related transcripts related to different pathways, including Toll, immune deficiency (IMD), c-Jun N-terminal kinase and JAK-STAT (electronic supplementary material, data sheet 1, file 2). The identified transcripts included various pattern recognition receptors (PRRs) that potentially interact with the mosquito’s microbiota, inducing the production of reactive oxygen species (ROS), AMPs and the phenoloxidase cascade system of melanization. The PMG expressed seven types of AMPs, namely cecropin A and A2, cecropin B and B1, cecropin N, defensin A and defensin B. Notably, the expression of CECA2 was higher in the PMG (figure *5e*).

The identified PRRs included a range of molecules such as peptidoglycan recognition protein (PGRP), C-type lectin, lysozymes, α-1,3-glucan-binding protein and ML-domain-containing protein. A total of 18 PGRP transcripts were identified, representing both secreted and membrane-bound receptors that recognize pathogen-derived molecules based on their structural composition [36]. Specifically, PGRP-1, -S1, -LB and -LC with isoforms A and B were identified, while only one type was found for the remaining PGRP receptors. PGRP-LC, -LB and -S1 (electronic supplementary material, data sheet 1, file 2) are known to activate the IMD and Toll pathways in mosquitoes in response to Gram-negative bacteria [37,38]. Carbohydrate-recognition proteins such as C-type lectin and C-type lectin-like domain proteins were also found in the PMG, highlighting their association with the immune function (figure 2, table 2).

The midgut of haematophagous insects can generate ROS in response to the presence of blood or microorganisms [39]. The level of ROS in the whole midgut of *Tx. theobaldi* was determined using the FOX and Griess methods to measure soluble peroxides (H_2_O_2_) and NO, respectively. A H_2_O_2_ level of 423.4 ± 4.619 µM was found, while NO was not detected.

To maintain redox balance and prevent oxidative stress in midgut epithelial cells, the involvement of antioxidant enzymes is crucial. In the PMG of *Tx. theobaldi*, 318 transcripts involved in metabolism detoxification were identified, including enzymes such as catalase (CAT), superoxide dismutase (SOD), cytochrome P450 and glutathione-S-transferase (electronic supplementary material, data sheet 1, file 2). The expression profiles of CAT and SOD, which are enzymes involved in redox homeostasis, were investigated using RT-qPCR. The expression levels of these genes were elevated in the AMG (figure *5f–g*).

Two transcripts coding for ferritin subunits, *Fer1HCH* and *Fer2LCH*, were detected in the PMG transcriptome. Fer2LCH was highly expressed in the AMG, but its expression was higher in PMG (figure *5h*). It is suggested that the elevated levels of ferritin, which detoxify, store and transport iron, serve as a reservoir during periods of metal scarcity [40,41].

### 3.7. Families of peptide precursor, molecule binding and hormone transport domains

Transcripts encoding putative precursor proteins for diuretic hormone, diuretic hormone 31, juvenile hormone (JH)-inducible protein, neuropeptide F, neuropeptide-like protein 31 and pigment-dispersing hormone were identified in the *Tx. theobaldi* midgut transcriptome (table 2; electronic supplementary material, data sheet 4, file 2). PMG also expresses the OBP family, which is seen in the chemosensory organs of insects. In addition, transcripts involved with intracellular metabolism of JH such as farnesoic acid 0-methyl transferase, farnesoic acid o-methyl transferase-like protein and epoxide hydrolase are also present in PMG (table 2; electronic supplementary material, data sheet 1/data sheet 4, file 2).

### 3.8. Molecular transport

Transcripts related to transporter and channel categories were abundant in PMG , including the subdivisions ADP/ATP translocase, glutamate/aspartate and neutral amino acid transporters; sugar transporter; and numerous subunits of vacuolar H+-ATPase, which were quite representative (approx. 41% of all transporters) (electronic supplementary material, data sheet 1, file 2), and Na^+^/K^+^-ATPase accounted for 2.93% of the contigs (electronic supplementary material, data sheet 1, file 2). It seems that most molecules can be transported across cell membranes in the PMG via membrane transport proteins, which are ∼approximately 86% of all transporters, with an emphasis on sodium-dependent transporters.

### 3.9. Microorganisms and transposable elements

The PMG expressed transcripts corresponding to coat protein, replication protein 1 a and movement protein, showing 98–100% identity with Brome mosaic virus (BMV). Additionally, some contigs encoding phage proteins, such as Tt-22239_FR2_672-12, were identified, which were 98% identical to enterobacteria phage S13 (electronic supplementary material, data sheet 1, file 2). These phage proteins may have originated from bacterial transcripts.

TE in PMG were highly diverse, although their expression levels were low (rmRPKM < 1) (table 2; electronic supplementary material, data sheet 1, file 2). A total of 195 transcripts were identified, with 51% belonging to class I (retrovirus-like elements) and 49% belonging to class II (transposons or DNA transposons). Among the 37 retrotransposons, the most abundant superfamilies in PMG were Bel, CR1 and Gypsy, which belong to class I. Additionally, eight contigs encoding retroviral proteins, including Gag, Pol and Env proteins, were identified. Interestingly, a transcript matching the retrovirus-related pol polyprotein from transposon TNT 1-94 found in *Nicotiana tabacum* (tobacco) was also identified. The most abundant class II elements were from the *tc/mariner* superfamily (43%).

## 4. Discussion

The midgut of *Tx. theobaldi* is divided into three segments: AMG1, AMG2 and PMG [12]. RNA-seq of the PMG region was carried out owing to its significance as the primary site for the synthesis of enzymes and components of the PM, digestion of complex biomolecules and the primary site of interactions with pathogens [42]. Our transcriptome analysis has highlighted the classes related to digestion and innate immune response owing to the crucial role of PMG in balancing digestive processes and immunity [43]. The expression pattern of most functional classes in the PMG of *Tx. theobaldi* was found to be similar to that observed in the PMG of *Ae. aegypti* and *An. gambiae* that were subjected to sugar-based diets [8,9], including the classes of peptidases (trypsin and chymotrypsin) and transcripts involved in translation. Additionally, the expression of representatives from the main functional classes present in the PMG was analysed, with a comparison made to the anterior region. Our data revealed that each midgut region plays a distinct role in digestion, highlighting differences in comparison with haematophagous mosquitoes.

### 4.1. Peritrophic matrix

In spite of its non-haematophagic diet, *Tx. theobaldi* produces a type I-like PM that is constitutively secreted by midgut cells [12] and composed of peritrophins with simple structures. On the contrary, in *An. gambiae*, the presence of several structurally more complex peritrophins might be interpreted as an adaptation to haematophagy. However, the acquisition of CDB domains by peritrophins most likely occurred after dipteran diversification and may be an adaptive evolution to the protease-rich environment [44,45]. On the other hand, the remarkable number of peritrophins reported in culicids, as observed in larvae, may be considered an adaptation to defense against pathogens, given that most larvae feed on organic matter rich in microorganisms. Therefore, it would be opportune to further investigate the role of peritrophins in the Culicidae family.

Another essential component of PM, the mucins, is characterized by a series of uninterrupted Pro/Thr/Ser residues referred to as mucin-like domain [46]. Among the mucins of *Tx. theobaldi*, the contribution from the secreted class was approximately 12%, and their presence reinforces the existence of a PM which has been previously detected in this species [12]. Secreted mucins are expected to prevent pathogens from accessing the midgut epithelium [46,47], and they also increase nutrient availability through enzyme immobilization in the ectoperitrophic space of the midgut [48].

### 4.2. Metabolism

Several sequences of α-amylase were identified in the PMG transcriptome of *Tx. theobaldi*, including the contig Tt-28334_FR3_60-659 co-orthologue, indicating that this diversity may represent an adaptive response to different plant-produced inhibitors [49]. Although α-amylases, glycoside hydrolases, glucose transporters and sucrose transporters are expressed in the PMG, the primary site for carbohydrate digestion is in the AMG regions 1 and 2. This concentration of transcripts encoding α-amylase in AMG is consistent with similar reports in adult *An. gambiae* [8]. Interestingly, in *Tx. theobaldi*, the α-amylase transcripts are organized into multiple clusters, with two distinct catalytic domains: the domain found in bacterial α-amylases and the domain found in maltases. This organization suggests a functional diversification that may increase the range of carbohydrates that can be efficiently digested by this species [49]. Additionally, chitinolytic enzymes, such as β-N-acetylhexosaminidase, have been detected in *Tx. theobaldi*. These enzymes may play a role in the formation and degradation of PM, which can affect the digestion of carbohydrates and other food components [50].

The α-glucosidase isoforms ab and C of *Tx. theobaldi* belong to family 13 of glycosyl-hydrolases (GH13). These enzymes facilitate the hydrolysis of glycosidic linkages in oligosaccharides and disaccharides. α-Glucosidase ab is associated with the membrane of the PMG in *Tx. theobaldi*. Other members of the GH13 family, such as maltase glucoamylase and β-glucosidase lactase phlorizin hydrolase, may play crucial physiological roles in carbohydrate digestion. Hixson et al. [9] also observed glucosidases in the PMG of *Ae. aegypti* and *An. gambiae* when fed with sugar. The acidity in the posterior region of *Tx. theobaldi* is ideal for the activity of α-glucosidases.

The PMG of *Tx. theobaldi* exhibited a wide range of transcripts with similar expression levels for serine protease and trypsin, consistent with previous studies on haematophagous mosquitoes fed on sugar [8,51,52]. For example, trypsin 4 resembles early trypsins observed in other mosquistoes [53,54]. This suggests that the midgut of *Tx. theobaldi* may secrete proteinases at a basal level to enhance digestion efficiency, as observed in other insects when the diet is nutritionally poor [55].

Our findings also confirmed the presence of trypsin activity in the whole midgut. In *Tx. theobaldi*, the synthesis of digestive proteases, including serine protease and trypsin, appears to be maintained at a basal level, reflecting the lower demand for proteolytic activity in non-haematophagous insects during digestion. This pattern mirrors the findings in adults of the non-haematophagous *Clogmia albipunctata* (Nematocera; Psychodidae). In this lower dipteran species, trypsin activity decreased following the ingestion of a protein-rich diet, including human serum [56]. It is plausible that a similar process is unfolding in *Tx. theobaldi*, warranting further investigation.

Interestingly, the presence of serine-protease orthologs in *Tx. theobaldi* and other analysed mosquitoes suggests that multiple duplication events occurred after speciation in Culicidae, implying that these proteases may have diverse functional roles in *Tx. theobaldi*.

The detection of cysteine proteases of the C1 family in the PMG of *Tx. theobaldi*’s midgut is a novel finding that has not been previously reported in mosquitoes. These cysteine peptidases may complement the action of serine proteases in luminal protein digestion. Furthermore, the isoforms of cathepsin B and cathepsin L secreted in the PMG of this mosquito have been shown to play a role in digestion and immune response in triatomines, hemipterans and cyclorrhaphous Diptera [57,58]. The detection of these proteases suggests that the posterior segment of the midgut exhibits an acidic environment, given that cysteine proteases are most effective in an acidic pH [59].

Our transcriptomic analysis revealed the presence of transcripts that are essential for lipid metabolism in *Tx. theobaldi*. Among the digestive lipases, PTLs were found to be distributed throughout the midgut of this mosquito and may play a crucial role in the hydrolysis of triacylglycerols (TAGs), especially in the PMG. Similar results were observed in *An. gambiae* [8], suggesting a conserved function of PTLs across different mosquito species. The metabolites derived from TAG hydrolysis by lipases and esterases are likely to be absorbed by enterocytes and converted into complex lipids [60]. Additionally, the presence of several transport proteins, such as apolipophorins, Niemann–Pick C disease protein and sterol carrier protein 2, suggests that there is intense lipid traffic between the midgut tissue and haemolymph. Sterols are essential for hormone production in insects and are typically obtained from their diet [61]. The route involving the digestion and metabolism of lipids detected in the midgut of *Tx. theobaldi* appears to be crucial for obtaining lipids from nectar, where lipids are present in trace amounts [62]. This finding suggests that the ability to efficiently digest and metabolize lipids may confer sufficient amounts of sterols and other essential nutrients by *Tx. theobaldi*.

### 4.3. Protease inhibitors

The majority of protease inhibitors detected in the PMG contigs of *Tx. theobaldi* belong to the serpin class, which plays a crucial role in the immune response of mosquitoes [63,64]. In *Tx. theobaldi*, the serpins secreted in the midgut lumen may regulate the activity of serine proteases and exogenous proteases from plants or pathogens. Our study also detected a Kazal-type serine protease inhibitor, which has been shown to reduce dengue virus (DENV) infection in *Ae. aegypti* [65]. Additionally, two other protease inhibitor proteins, thyroglobulin and cystatin, were identified in the PMG of *Tx. theobaldi*, which are potentially involved in the innate immune response of mosquitoes [66,67]. Our findings suggest that *Tx. theobaldi* employs a diverse array of protease inhibitors to regulate protease activity in the midgut and possibly aid defense against pathogens. Further studies are needed to investigate the precise mechanisms by which these inhibitors function in the midgut of *Tx. theobaldi*.

### 4.4. Hormones

Our transcriptomic analysis identified the presence of peptide groups in the PMG of *Tx. theobaldi*, indicating their potential involvement in the regulation of digestion. Among these, Neuropeptide F is a member of the neuropeptide Y family and has been reported in various insect species, including *Ae. aegypti* larvae [68] and *An. gambiae* [69]. Besides its role in regulating insect behaviour and physiology, such as feeding and metabolism [70], neuropeptide F is also detected in the midgut of adult female *Tx. theobaldi*, suggesting its potential involvement in feeding regulation in this mosquito species.

The OBP family was found to be expressed in the PMG and is also present in other non-sensory organs of insects, such as the proboscis, acting as solubilizers of hydrophobic nutrients [71]. One member of this family, juvenile hormone-inducible protein, was detected in PMG and is known to occur in the haemolymph of several mosquitoes [72]. In *Locusta migratoria*, this protein has been found to participate in the control of digestion [73]. Our study also identified other transcripts involved in the intracellular metabolism of JH, suggesting the possibility of its synthesis and action in gut cells, as seen in the gut of adult *Drosophila* [74].

### 4.5. Immunity and detoxification

The expression of members of the PGRP family suggests their role in activating several immune pathways, including IMD and Toll pathways that induce the synthesis of AMPs and activate a prophenoloxidase cascade in *Tx. theobaldi*. In addition to PGRP, lysozyme is another enzyme with antimicrobial activity in the mosquito midgut, with both P-type and C-type lysozymes observed. The activity of lysozyme isoforms C and P have been reported in other mosquitoes [75–77]. Several members of the CTL family were also observed in the PMG, and they may integrate the mucin-rich mucus layer and play a role in antibacterial, antiplasmodial and antiviral responses in mosquitoes [78–80]. Finally, the presence of β-1,3-glucan-binding protein suggests its involvement in antifungal immunity, similar to what has been observed in *Ae. aegypti* [36].

The AMPs such as cecropin and defensin exhibit broad-spectrum activity against various pathogenic bacteria, fungi, protozoa and viruses. They can act by destabilizing the cell membrane or targeting intracellular components [81]. Notably, the expression of cecropin A2 was found to be significantly higher in the PMG compared with AMG 1 and 2. Furthermore, its presence has also been detected in the AMG of *An. gambiae* [8,82].

Cytochrome P450 (CYP450) is one of the predominant enzymes found in the PMG responsible for detoxification and has been linked to insecticide resistance in mosquitoes [83,84]. The diversity of CYP450 subfamilies observed in *Tx. theobaldi* is likely associated with chemical defense against environmental xenobiotics present in their diet or produced by pathogens. In addition, it was detected high expression levels of CAT and SOD in the AMG, which can help prevent damage induced by ROS that often affects various cell functions, including cell differentiation and apoptosis [85]. The midgut of female *An. aquasalis* also showed high levels of CAT transcripts [86].

The production of epithelial NO by NO synthase (NOS) is a potential host defense against pathogens, such as *Plasmodium* [87]. Notably, no transcript of NOS was detected in the midgut of *Tx. theobaldi*. The absence of transcripts for the NOS enzyme of this mosquito and the competition of L-arginine by the polyamine biosynthesis pathway limits NOS transcription [87].

### 4.6. Viral and transposable elements

The presence of complete viral sequences in the PMG, which matched BMV, a plant pathogen belonging to the alphavirus-like superfamily, supports the hypothesis of horizontal gene transfer from virus to mosquito [88,89]. Additionally, we identified the transposon TNT 1-94 from tobacco, which is involved in different plant responses to external stimuli [90].

TE, such as transposons, are mobile genetic elements that can increase variability within organisms [91]. The transcripts encoding TE in the PMG exhibited high diversity, but with lower levels of expression (rmRPKM < 1). Interestingly, unlike in the transcriptome of *Anopheles funestus* [92], the most abundant TE in *Tx. theobaldi* were class II or DNA transposons, with a large number of transcripts coding for transposases of the *tc/mariner* superfamily. This suggests that active transposition of these elements may be occurring in the mosquito transcriptome.

### 4.7. Transport

After digestion of sugars, transporters are required to move the resulting nutrients to both the midgut epithelium and the body cavity. In the PMG of *Tx. theobaldi*, transcripts of sugar transporters are present, suggesting that the absorption of sugar digestion derivatives also occurs in this region. The acidic character of the apocrine secretion in PMG of *Tx. theobaldi* is probably owing to the presence of H+-ATPase and Na+/K+-ATPase in the apical membrane of the PMG [12], a feature also found in non-haematophagous *Cl. albipunctata* [56] and *Ae. aegypti* [93]. The vacuolar H+-ATPase subunits can participate in potassium ion transport, which causes alkalinity [94]. Furthermore, many amino acid transporters were also observed in the PMG of *Tx. theobaldi*, suggesting that the digestion of various proteins and absorption of amino acids predominantly occur in this region, similar to other mosquitoes [9]. Efficient use of transporters is crucial for phytophagous insects like *Tx. theobaldi* to obtain maximum nutritional benefit [95].

## 5. Final considerations

The analysis of the transcriptome of PMG, combined with RT-qPCR and enzymatic activity in *Tx. theobalbi*, has led to significant insights into the gene expression patterns within the midgut of this species. Our study is the first to reveal detailed information about gene expression in the midgut of an autogenous mosquito, which is believed to be a retained trait from its non-haematophagous ancestor, according to previous research [96–98].

Several transcripts typically associated with haematophagous mosquitoes, such as serine endopeptidases, were identified through the analyses of the transcriptome of the PMG of *Tx. theobaldi*. The regionalization of the midgut into three distinct segments, AMG1, AMG2 and PMG, was also confirmed for several important classes of transcripts, including protein metabolism, carbohydrate metabolism, lipid metabolism, immune response and PM. Interestingly, this mosquito constitutively produces proteins, such as trypsins and peritrophins, that are typically modulated during blood digestion in other mosquitoes, suggesting possible structural and functional differences.

TE can play a significant role in the evolutionary process of this insect. We observed, for example, retrovirus-related pol polyprotein transposon from TNT 1-94 associated with tobacco, potentially mediating horizontal gene transfer between the virus that affects plants and *Tx. theobaldi*. In addition, the absence of sequences related to the families of viruses that occur in vector mosquitoes suggests the association of this mosquito with a sugar-based diet in the past. With this study, we hope to contribute to filling in the gaps in the evolution of the eating habits of the Culicidae family, which is mainly composed of information about vector mosquitoes.

## Data Availability

RNA sequences: Genbank accessions GKNE01000001. The datasets supporting this article have been uploaded at: https://www.ncbi.nlm.nih.gov/sra/PRJNA983392. Supplementary material is available online [99].
